# MicroRNA-532-5p protects against cerebral ischemia-reperfusion injury by directly targeting CXCL1

**DOI:** 10.18632/aging.202846

**Published:** 2021-04-18

**Authors:** Yuanyuan Shi, Zhongquan Yi, Panwen Zhao, Yun Xu, Pinglei Pan

**Affiliations:** 1 Department of Neurology and Central Laboratory, The Yancheng School of Clinical Medicine of Nanjing Medical University, Yancheng 224001, Jiangsu, China; 2 Department of Neurology, Drum Tower Hospital, Medical School of Nanjing University, Nanjing 210008, Jiangsu, China; 3 Department of Central Laboratory, The Yancheng School of Clinical Medicine of Nanjing Medical University, Yancheng 224001, Jiangsu, China

**Keywords:** miR-532-5p, CXCL1, CXCR2, NF-κB

## Abstract

We investigated the function of microRNA (miR)-532-5p in cerebral ischemia-reperfusion injury (CI/RI) and the underlying mechanisms using oxygen-glucose deprivation and reperfusion (OGD/R)-treated SH-SY5Y cells and middle cerebral artery occlusion (MCAO) model rats. MiR-532-5p levels were significantly downregulated in OGD/R-treated SH-SY5Y cells and the brain tissues of MCAO model rats. MiR-532-5p overexpression significantly reduced apoptosis, reactive oxygen species (ROS), and inflammation in the OGD/R-induced SH-SY5Y cells. Bioinformatics analysis using the targetscan and miRDB databases as well as dual luciferase reporter assays confirmed that miR-532-5p directly binds to the 3’UTR of C-X-C Motif Ligand 1 (CXCL1). Methylation-specific PCR (MSP) analysis showed that miR-532-5p expression was reduced in OGD/R-treated SH-SY5Y cells because of miR-532-5p promoter hypermethylation. Moreover, 5-azacytidine, a methylation inhibitor, restored miR-532-5p expression in OGD/R-treated SH-SY5Y cells. Brain tissues of MCAO model rats showed significantly increased cerebral infarction areas, cerebral water, neuronal apoptosis, and activated CXCL1/CXCR2/NF-κB signaling, but these effects were alleviated by intraventricular injection of miR-532-5p agomir. These findings demonstrate that miR-532-5p overexpression significantly reduces *in vitro* and *in vivo* CI/RI by targeting CXCL1. Thus, miR-532-5p is a potential therapeutic target for patients with CI/RI.

## INTRODUCTION

Stroke is a form of brain injury due to cerebral vascular rupture or interruption in blood supply to the affected part of the brain, and it leads to long-term disability in nearly 40 million individuals world-wide every year [1, 2]. In several cases, restoration of cerebral blood supply is associated with cerebral ischemia-reperfusion injury (CI/RI) [3, 4]. The currently available treatments for CI/RI include surgery and drugs, but the efficacy of these treatments is limited [5, 6]. Therefore, there is an urgent need for newer and more effective treatment strategies for CI/RI.

MicroRNAs (miRNAs) are non-coding RNAs (22 nucleotides in length) which can modulate mRNA expression [7, 8]. Several studies have shown that dysregulation of specific miRNAs influences CI/RI progression. For example, Liu *et al*. reported that miR-211 protected against CI/RI in the MCAO model rats by downregulating p53-upregulated modulator of apoptosis (PUMA) [9]. MiR-193b-3p protected against focal CI/RI injury by inhibiting 5-LOX expression [10]. Furthermore, miR-532-5p attenuates the progression of ischemic stroke through inactivating PTEN and upregulating PI3K/Akt signaling pathway [11, 12]. However, the detailed mechanisms underlying the neuroprotective effects of miR-532-5p in CI/RI are not fully clear.

Chemokine (CXC motif) ligand 1 (CXCL1) is known to be an important chemoattractant via interacting with G-protein-coupled receptor chemokine (CXC motif) receptor 2 (CXCR2) [13]. In addition, CXCL1 could modulate inflammation and cancer progression [14]. Meanwhile, CXCL1 levels are upregulated in hypoxic-Ischemic brain Injury [15]; however, the association between miR-532-5p and CXCL1 in CI/RI is unclear.

Therefore, this study aimed to investigate the mechanisms underlying the neuroprotective role of miR-532-5p during CI/RI using SH-SY5Y cells grown under oxygen-glucose deprivation and reperfusion (OGD/R) conditions and the cerebral artery occlusion (MCAO) rat model.

## RESULTS

### MiR-532-5p overexpression reduces apoptosis in OGD/R-treated SH-SY5Y cells

**Figure 1:**
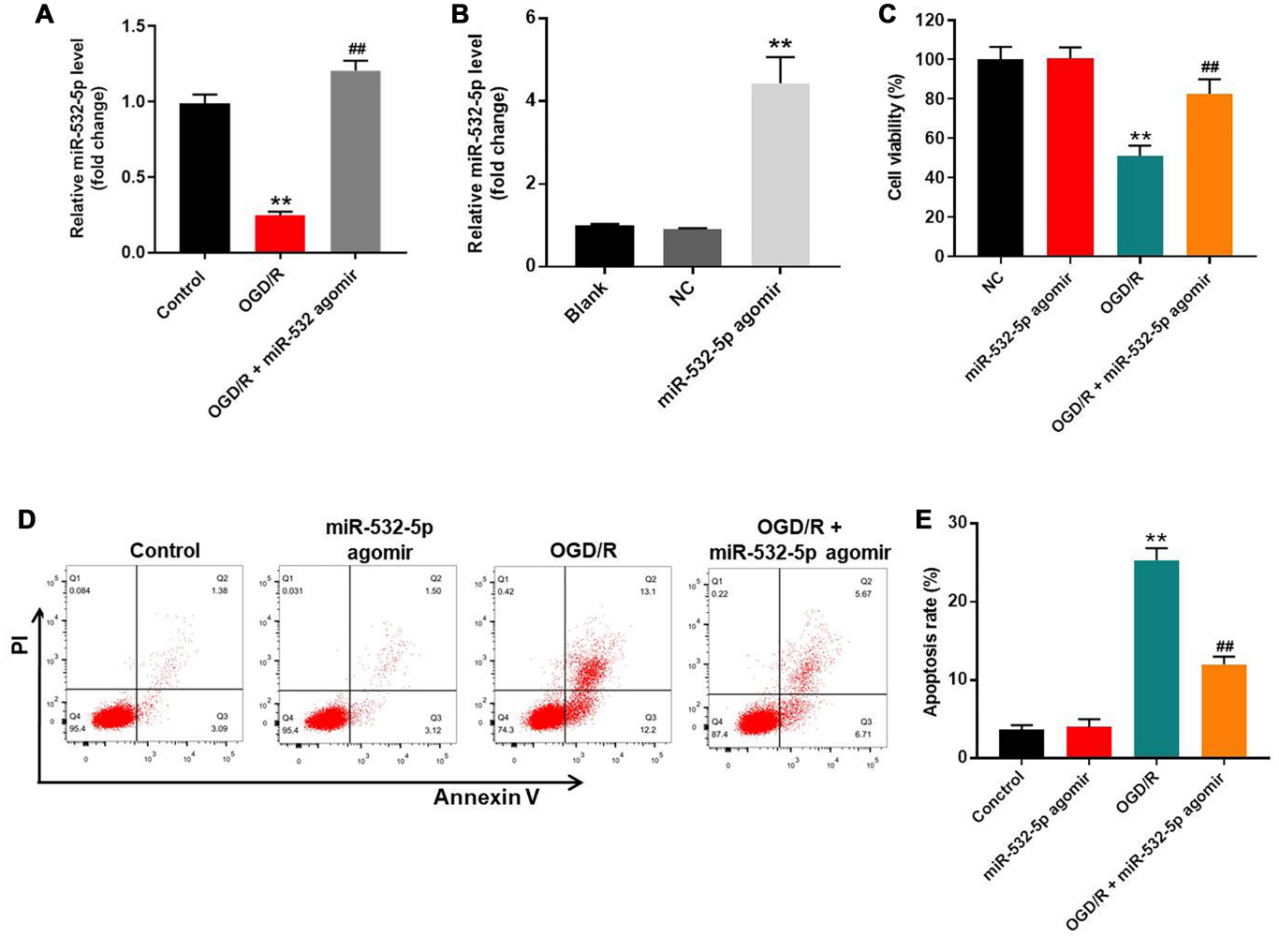
**MiR-532-5p overexpression significantly reduces OGD/R-induced apoptosis in SH-SY5Y cells.** (**A**) RT-qPCR analysis shows miR-532-5p levels in control and miR-532-5p agomir-transfected SH-SY5Y cells treated with or without OGD/R. (**B**) RT-qPCR analysis shows miR-532-5p levels in control and miR-532-5p agomir-transfected SH-SY5Y cells. (**C**) CCK-8 assay analysis shows the viability of control and miR-532-5p agomir-transfected SH-SY5Y cells treated with or without OGD/R. (**D**) Flow cytometry analysis of Annexin V plus propidium iodide (PI) stained control and miR-532-5p agomir-transfected SH-SY5Y cells treated with or without OGD/R. (**E**) Quantitative analysis of FACS data shows the percentage of apoptotic cells (Annexin-V+ PI+ plus Annexin-V+ PI+) in control and miR-532-5p agomir-transfected SH-SY5Y cells treated with or without OGD/R. ^**^P < 0.01 compared to control; ^##^*P* < 0.01 compared to OGD/R. All experiments were performed in triplicate.

We used OGD/R-treated SH-SY5Y cells as an *in vitro* model of CI/RI. The levels of miR-532-5p were significantly reduced in OGD/R-treated SH-SY5Y cells compared to the corresponding controls, but remained notably higher in miR-532-5p agomir-transfected SH-SY5Y cells under treatment of OGD/R (Figure 1A). Moreover, miR-532-5p levels were upregulated in the miR-532-5p agomir-transfected SH-SY5Y cells than the corresponding controls (Figure 1B). CCK-8 assay results showed that OGD/R reduced the viability of SH-SY5Y cells, whereas, miR-532-5p overexpression increased OGD/R-treated SH-SY5Y or PC-12 cell viability (Figure 1C and Supplementary Figure 1A). Furthermore, miR-532-5p overexpression markedly decreased OGD/R-induced apoptosis in SH-SY5Y or PC-12 cells (Figure 1D–1E and Supplementary Figure 1B). To sum up, these results demonstrated that miR-532-5p overexpression significantly decreased apoptosis of OGD/R-induced SH-SY5Y cells.

### MiR-532-5p overexpression significantly reduced oxidative stress in OGD/R-treated SH-SY5Y cells

**Figure 2:**
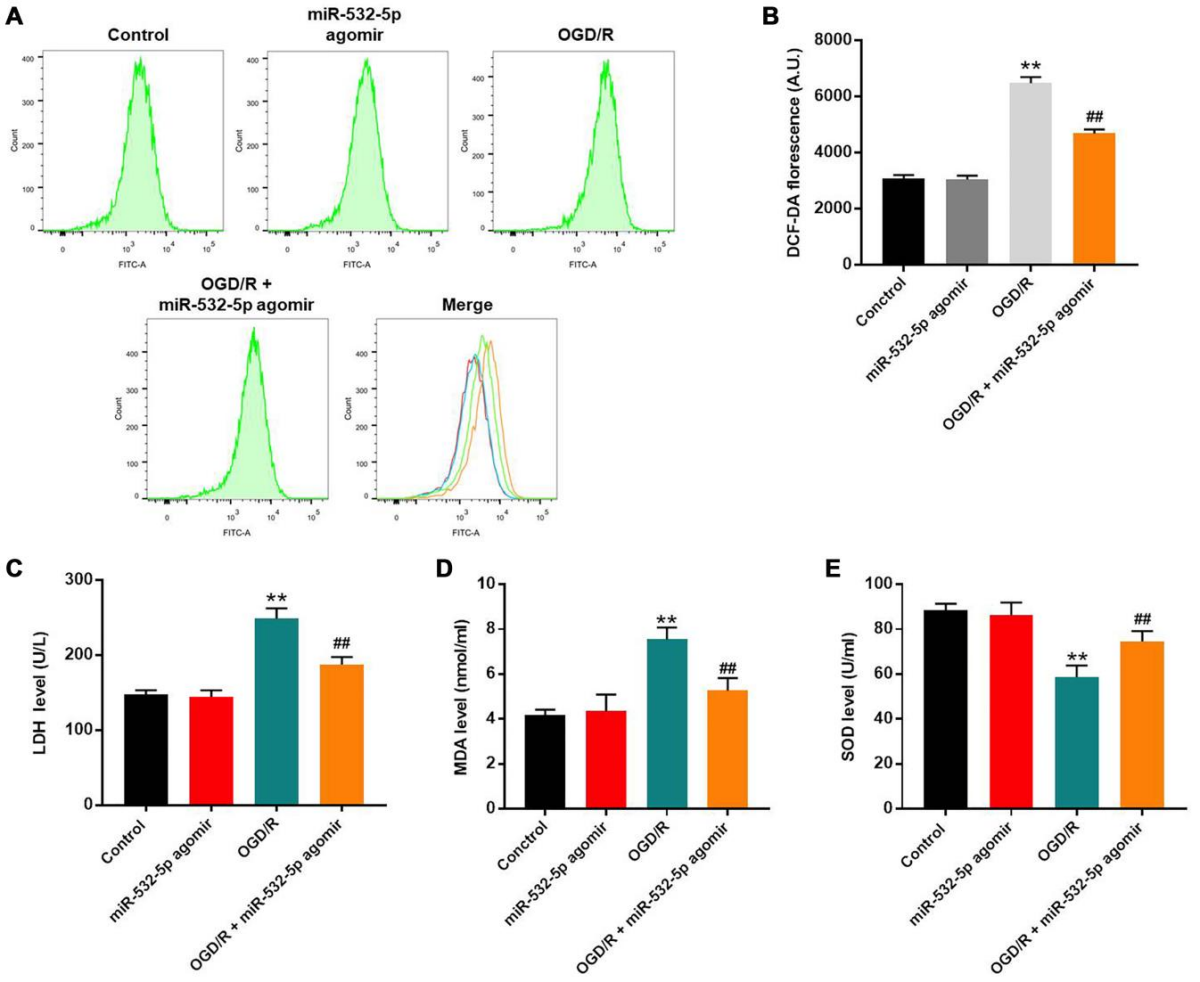
**MiR-532-5p overexpression significantly reduces OGD/R-induced oxidative stress in SH-SY5Y cells.** (**A**) FACS plots show ROS levels based on DCF-DA fluorescence intensity in control and miR-532-5p agomir-transfected SH-SY5Y cells treated with or without OGD/R. (**B**) The histogram plots show the levels of DCF-DA fluorescence intensity in control and miR-532-5p agomir-transfected SH SY5Y cells treated with or without OGD/R. (**C**–**E**) The histogram plots show the concentrations of (**C**) LDH, (**D**) MDA and (**E**) SOD in the supernatants of control and miR-532-5p agomir-transfected SH-SY5Y cells treated with or without OGD/R, as analyzed using ELISA kits. ^**^P < 0.01 compared to control; ^##^*P* < 0.01 compared to OGD/R. All experiments were performed in triplicates.

Flow cytometry analysis showed that ROS levels were significantly increased in SH-SY5Y cells upon OGD/R treatment, but were reduced by miR-532-5p over-expression (Figure 2A–2B). Furthermore, LDH and MDA levels were higher in the supernatants of OGD/R-treated SH-SY5Y cells than untreated controls, but were significantly reduced by miR-532-5p over-expression (Figure 2C–2D). Moreover, SOD levels were decreased in the supernatant of OGD/R-treated SH-SY5Y cells than the corresponding controls, but were higher in miR-532-5p over-expressing SH-SY5Y cells treated with OGD/R (Figure 2E). Taken together, these demonstrated that miR-532-5p upregulation inhibited OGD/R-induced oxidative stress.

### MiR-532-5p overexpression decreased the levels of pro-inflammatory cytokines in the OGD/R-induced SH-SY5Y cells

**Figure 3:**
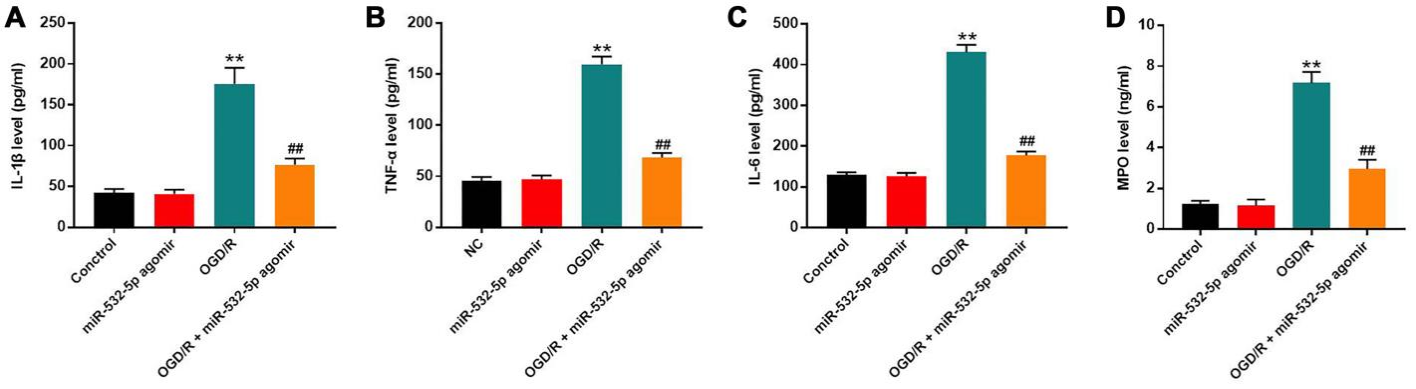
**MiR-532-5p overexpression significantly inhibited OGD/R-induced inflammation in SH-SY5Y cells.** (**A**–**D**) ELISA assay results show the levels of (**A**) IL-1β, (**B**) TNF-α, (**C**) IL-6, and (**D**) MPO in the supernatants of control and miR-532-5p agomir-transfected SH-SY5Y cells treated with or without OGD/R. ^**^P < 0.01 compared to the control; ^##^*P* < 0.01 compared to OGD/R. All experiments were performed in triplicate.

We performed ELISA assays to estimate the secretion of pro-inflammatory cytokines in the supernatants of control and miR-532-5p overexpressing SH-SY5Y cells treated with or without OGD/R. The levels of IL-6, TNF-α, IL-1β and MPO were markedly higher in the supernatants of OGD/R-treated SH-SY5Y cells than the controls, but were significantly reduced in miR-532-5p overexpressing SH-SY5Y cells treated with OGD/R (Figure 3A–3D). This suggested that miR-532-5p overexpression significantly inhibited the inflammatory response in SH-SY5Y cells.

### MiR-532-5p directly targets CXCL1

**Figure 4:**
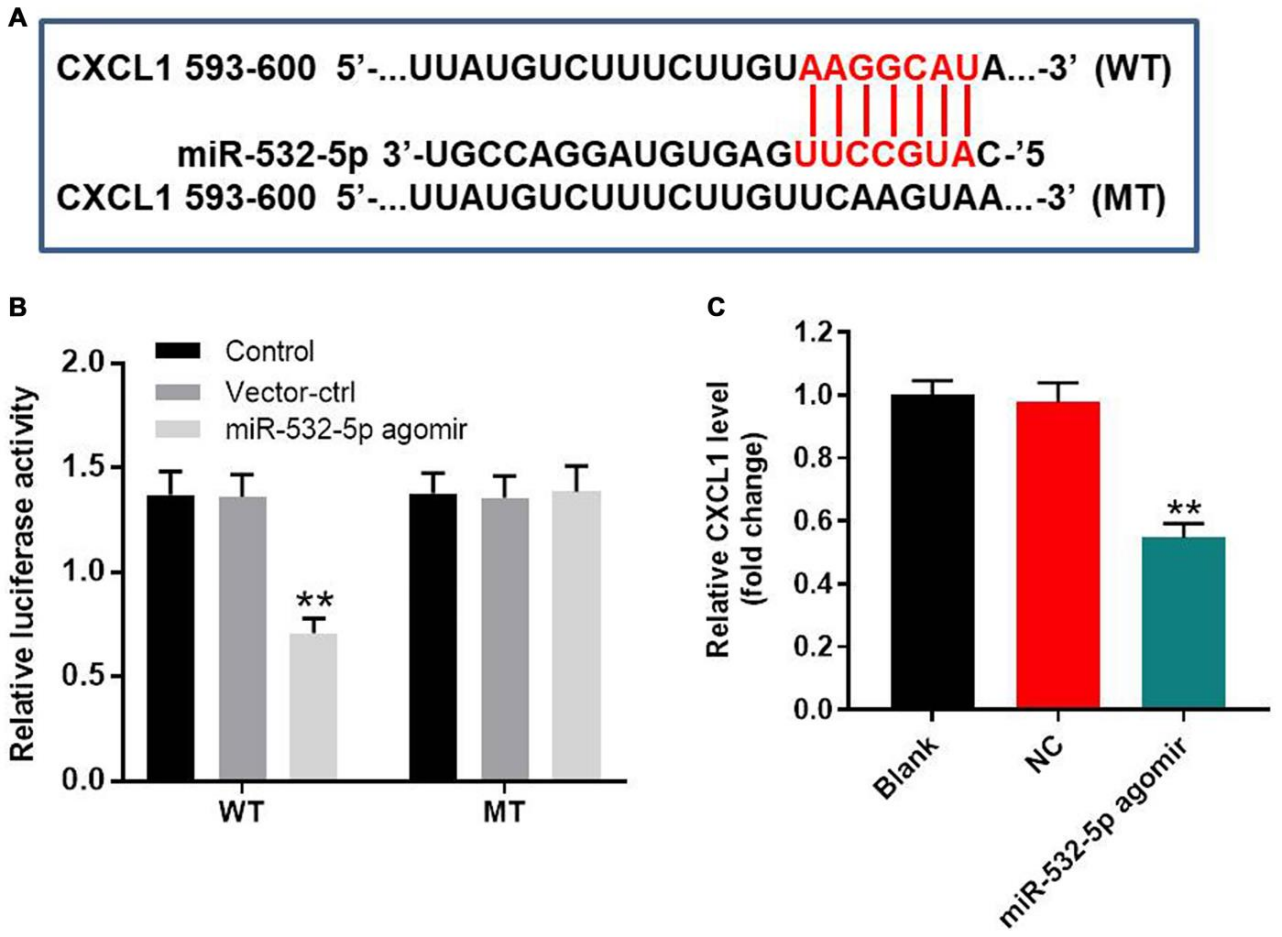
**MiR-532-5p directly targets CXCL1.** (**A**) A portion of the 3’UTR sequence of CXCL1 from nucleotides 593-600 represents the predicted target binding site for miR-532-5p. (**B**) Dual luciferase reporter assay results show the relative luciferase activity in SH-SY5Y cells co-transfected with miR-532-5p agomir and luciferase vector carrying wild-type (WT) or mutant (MT) CXCL1 3′-UTR. (**C**) RT-qPCR analysis shows the relative expression of CXCL1 in control and miR-532-5p agomir-transfected SH-SY5Y cells. ^**^P < 0.01 compared to control. All experiments were performed in triplicate.

We searched the Targetscan and miRDB databases and predicted CXCL1 as the potential target of miR-532-5p (Figure 4A). MiR-532-5p mimics greatly decreased the relative luciferase activity in SH-SY5Y cells co-transfected with vector cloned with wild-type 3’UTR of CXCL1 (WT-CXCL1), whereas, miR-532-5p mimics did not reduce the luciferase activity in SH-SY5Y cells co-transfected vector cloned with mutant 3’UTR of CXCL1 (MT-CXCL1) (Figure 4B). The level of CXCL1 mRNA was markedly downregulated in miR-532-5p-overexpressing SH-SY5Y cells (Figure 4C). These data demonstrated that miR-532-5p directly targeted CXCL1.

### OGD/R treatment increased methylation of the miR-532-5p promoter

**Figure 5:**
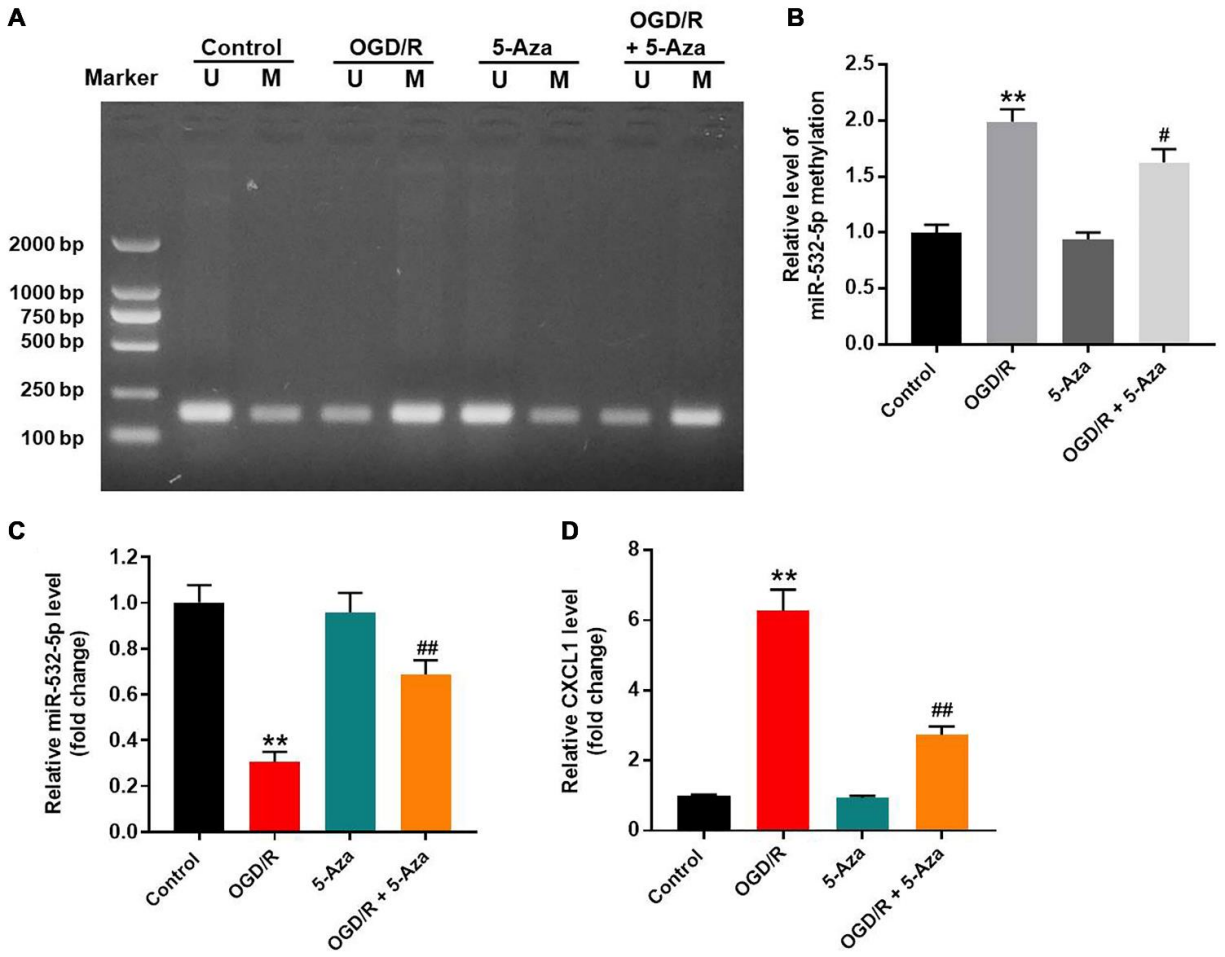
**OGD/R treatment increased methylation of the miR-532-5p promoter.** (**A**, **B**) Methylation-specific PCR (MSP) results show the methylation status of the CpG islands in the miR-532-5p promoter sequence in SH-SY5Y cells treated with OGD/R, 5-Aza or OGD/R + 5-Aza and untreated controls. ‘U’ refers to PCR with primers specific to unmethylated sequence and ‘M’ refers to PCR with primers specific to methylated sequence. (**C**) RT-qPCR analysis shows the relative expression levels of miR-532-5p in SH-SY5Y cells treated with OGD/R, 5-Aza or OGD/R + 5-Aza and untreated controls. (**D**) RT-qPCR analysis shows the relative expression levels of CXCL1 mRNA in SH-SY5Y cells treated with OGD/R, 5-Aza or OGD/R + 5-Aza compared to untreated controls. ^**^P < 0.01 compared to control; ^#^*P* < 0.05, ^##^*P* < 0.01 compared to OGD/R. All experiments were performed in triplicate.

Methylation-specific PCR (MSP) was applied to estimate the methylation status of miR-532-5p. OGD/R treatment significantly increased methylation of the miR-532-5p promoter, but these effects were partially reversed by 5-Azacytidine (5-Aza), a methylation inhibitor (Figure 5A–5B). RT-qPCR analysis showed that OGD/R treatment reduced miR-532-5p expression and upregulated CXCL1 mRNA levels in SH-SY5Y cells, but this phenomenon were inhibited by 5-Azacytidine treatment (Figure 5C–5D). These results demonstrated that OGD/R reduced miR-532-5p levels by increasing miR-532-5p promoter methylation.

### MiR-532-5p overexpression decreased OGD/R-induced inflammation via CXCL1/CXCR2/NF-κB axis

**Figure 6:**
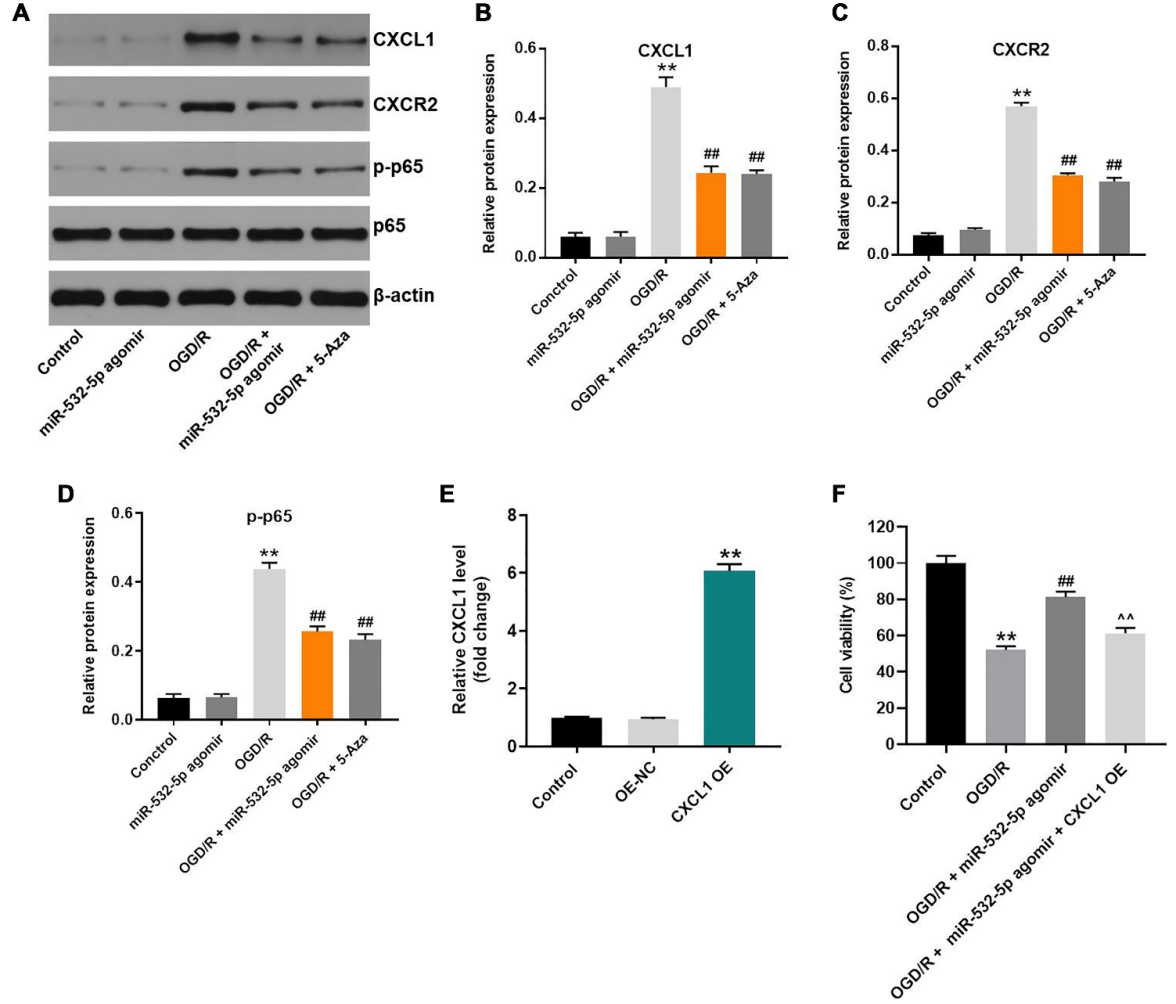
**MiR-532-5p overexpression suppresses OGD/R-induced inflammation in SH-SY5Y cells via CXCL1/CXCR2/NF-κB axis.** (**A**) Representative western blot shows the expression of CXCL1, CXCR2, p65 and p-p65 proteins in control or miR-532-5p agomir-transfected SH-SY5Y cells, treated with or without OGD/R. (**B**–**C**) The histogram plots show the expression levels of CXCL1 and CXCR2 proteins relative to β-actin protein levels in control or miR-532-5p agomir-transfected SH-SY5Y cells treated with or without OGD/R. (**D**) The histogram plot shows the levels of p-p65 relative to p65 levels in control or miR-532-5p agomir-transfected SH-SY5Y cells treated with or without OGD/R. (**E**) SH-SY5Y cells were transfected with NC or pcDNA3.1-CXCL1. The efficiency of cell transfection was detected by RT-qPCR. (**F**) The viability of SH-SY5Y cells was tested by CCK-8 assay. ^**^P < 0.01 compared to control; ^##^*P* < 0.01 compared to OGD/R. ^^^^*P* < 0.01 compared to OGD/R plus miR-532-5p agomir group. All experiments were performed in triplicate.

Next, we explored the mechanism by which miR-532-5p mediates CI/RI progression. The data showed that OGD/R upregulated the levels of CXCL1, CXCR2 and p-p65 proteins in SH-SY5Y cells, but this phenomenon was reversed by miR-532-5p over-expression or 5-Azacytidine treatment (Figure 6A–6D). Meanwhile, CXCL1 level was significantly upregulated by pcDNA3.1-CXCL1, and the effect of miR-532-5p on viability of OGD/R-treated SH-SY5Y cells was notably reversed by CXCL1 overexpression (Figure 6E–6F). These results suggested that miR-532-5p overexpression reversed OGD/R-induced inflammation through the CXCL1/CXCR2/NF-κB axis.

### MiR-532-5p reduces MCAO-induced brain pathology in the *in vivo* rat CI/RI model

**Figure 7:**
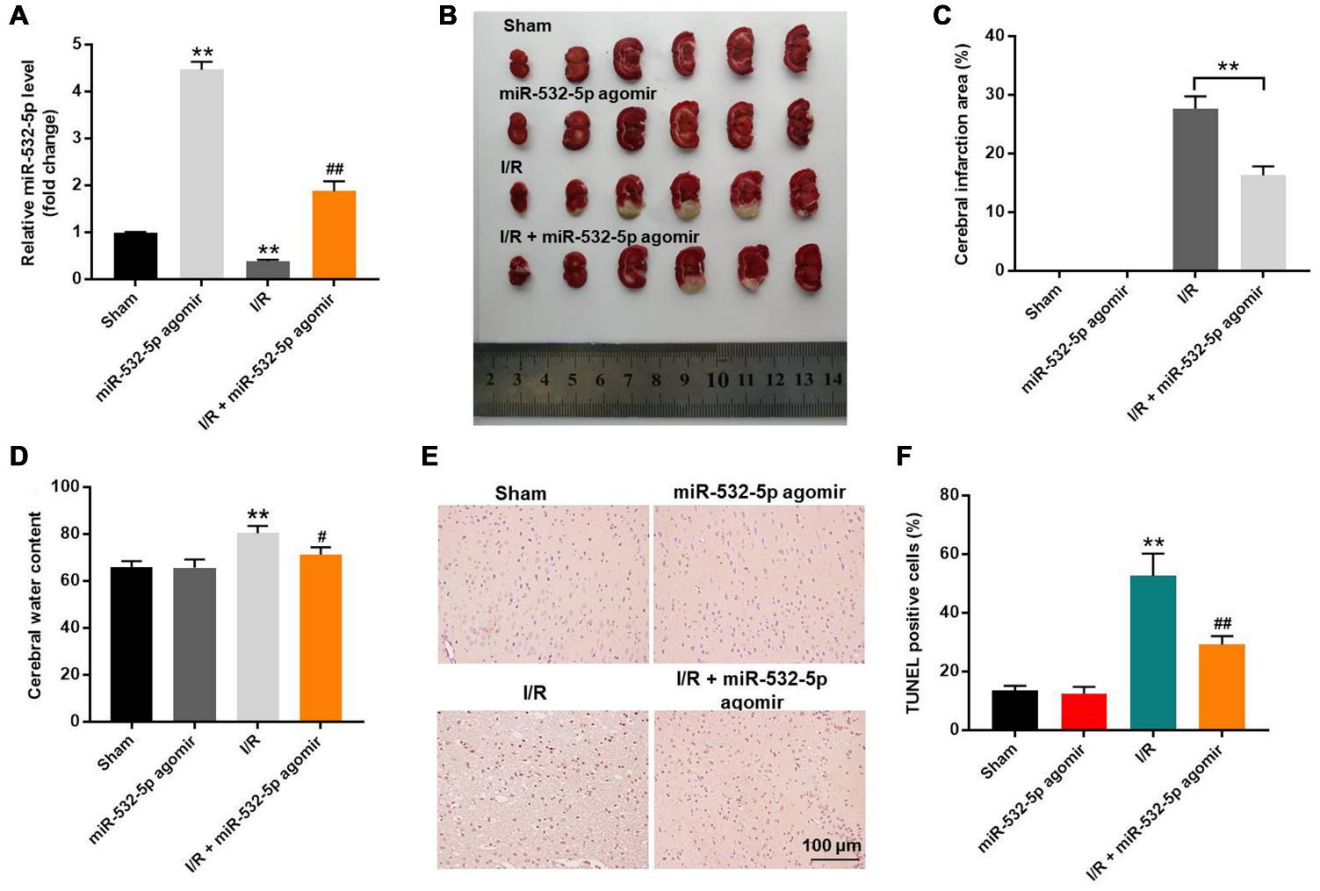
**Overexpression of miR-532-5p reduces brain pathology in the *in vivo* CI/RI model rats.** (**A**) RT-qPCR analysis shows the relative expression levels of miR-532-5p in the rat cerebral tissues in sham, miR-532-5p agomir, I/R and I/R plus miR-532-5p agomir groups. (**B**) Representative images show the brain tissues from sham, miR-532-5p agomir, I/R and I/R plus miR-532-5p agomir group rats. (**C**) Histogram plot shows cerebral infraction area based on TTC staining in sham, miR-532-5p agomir, I/R, and I/R plus miR-532-5p agomir group rats. ^**^P < 0.01 compared to sham. (**D**) The histogram plot shows cerebral water content in sham, miR-532-5p agomir, I/R and I/R plus miR-532-5p agomir group rats. (**E**, **F**) TUNEL staining results show the percentage of TUNEL-positive apoptotic neurons in the brain sections of sham, miR-532-5p agomir, I/R, and I/R plus miR-532-5p agomir group rats. ^**^P < 0.01 compared to Sham; ^#^*P* < 0.05, ^##^*P* < 0.01 compared to I/R. Each group consisted of 12 rats.

**Figure 8:**
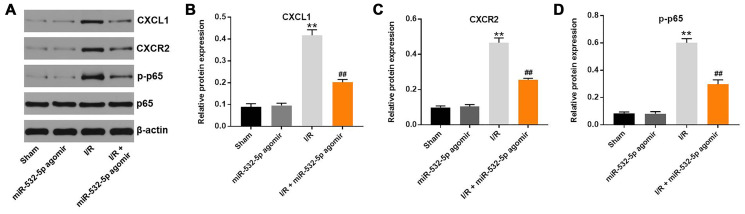
**MiR-532-5p overexpression inhibits in vivo CI/RI through the CXCL1/CXCR2/NF-κB signaling pathway.** (**A**) Representative western blot shows the expression levels of CXCL1, CXCR2, p65 and p-p65 proteins in brain tissues of sham, miR-532-5p agomir, I/R, and I/R plus miR-532-5p agomir group rats. (**B**–**C**) The histogram plots show the levels of CXCL1 and CXCR2 proteins relative to β-actin levels in brain tissues of sham, miR-532-5p agomir, I/R, and I/R plus miR-532-5p agomir group rats. (**D**) The histogram plot shows the levels of p-p65 relative to p65 in brain tissues of sham, miR-532-5p agomir, I/R, and I/R plus miR-532-5p agomir group rats. ^**^P < 0.01 compared to Sham; ^##^*P* < 0.01 compared to I/R. All experiments were performed in triplicates.

Finally, we established the *in vivo* middle cerebral artery occlusion (MCAO) rat model to determine the function of miR-532-5p in CI/RI. MiR-532-5p level was notably higher in miR-532-5p agomir group (Figure 7A). MiR-532-5p levels were reduced in the brain tissues of MCAO group rats (*P* < 0.01), but were higher in the miR-532-5p agomir plus MCAO group (Figure 7A). Furthermore, MCAO treatment significantly increased cerebral infarction area and cerebral water, but these effects were significantly reduced by miR-532-5p overexpression (Figure 7B–7D). Moreover, TUNEL assay showed that MCAO treatment significantly increased apoptosis in the rat brain tissues, but these effects were markedly decreased by miR-532-5p overexpression (Figure 7E–7F). The levels of CXCL1, CXCR2 and p-p65 proteins were significantly upregulated in the brain tissues of the MCAO group rats, but these effects were reversed by miR-532-5p overexpression (Figure 8A–8D). Taken together, these data demonstrated that miR-532-5p significantly reduced brain pathology in the rat model of CI/R.

## DISCUSSION

MicroRNAs are involved in multiple human diseases, and their dysregulation plays a critical role in CI/RI progression [9, 16]. This research demonstrated that miR-532-5p significantly reduced apoptosis in OGD/R-induced SH-SY5Y cells and MCAO-induced brain pathology in the CI/RI model rats. Previous reports revealed that miR-532-5p played a neuroprotective role during ischemic stroke [11, 12]. Our data were in line with these recent findings and further confirmed that miR-532-5p plays a protective role in CI/R. In contrast, a previous study suggested that miR-532-5p upregulation promoted proliferation of breast cancer cells [17]. This suggests contrasting roles for miR-532-5p in different cell types. Furthermore, OGD/R treatment increased miR-532-5p promoter methylation, thereby reducing miR-532-5p levels. Promoter methylation-mediated downregulation of miRNAs is commonly observed in tumorigenesis. For example, Ni et al. reported promoter methylation-associated downregulation of miR-638 in endometrial carcinoma [18]. Our findings are consistent with these results. Furthermore, our results confirm that OGD/R downregulated miR-532-5p level in SH-SY5Y cells by increasing miR-532-5p promoter methylation.

MicroRNAs regulate biological functions by decreasing expression of their target genes [19, 20]. This research confirmed that CXCL1 mRNA was the target of miR-532-5p in CI/RI-induced SH-SY5Y cells. CXCL1 is over-expressed in several human diseases and is a member of the chemotactic superfamily [21, 22]. CXCL1 promotes inflammation and progression of several cancers [23]. Our study demonstrated that CXCL1 promotes CI/RI progression. Furthermore, our findings show that miR-532-5p reduces CI/RI progression by targeting CXCL1. Wang et al. indicated that miR-532-5p inhibited glioma cell proliferation by targeting CSF1 [24].

CXCL1/CXCR2 (C-X-C motif chemokine receptor 2) mediated signaling promotes progression of inflammatory responses [25]. Manjavachi et al. reported that CXCL1 and CXCR2 suppressed the progression of peripheral neuropathy in rats [26]. Our study demonstrated that upregulation of miR-532-5p suppressed CI/RI through the CXCL1/CXCR2 pathway. CXCL1 is known to be a crucial mediator in inflammation and organ injury [27], and CXCR2 is the downstream protein of CXCL1 [28, 29]. In a previous study, CXCL1/CXCR2 pathway could lead to the injury of brain tissues [15]. Our research was consistent to the previous reports, suggesting that miR-532-5p could reverse OGD/R-induced cell growth inhibition via mediation of CXCL1/CXCR2 pathway. On the other hand. We also found that miR-532-5p inactivated NF-κB, which is a key mediator of tumorigenesis and inflammatory responses [28, 30]. Moreover, p65, a key protein in NF-κB signaling, and it is a crucial player in nervous and immune systems [31]. Ni *et al*. reported that the crosstalk between NFkB-dependent astrocytic CXCL1 and neuronal CXCR2 played a key role in chronic pain [32]. Our research confirmed that miR-532-5p overexpression alleviated CI/RI pathology in rat brains by inhibiting CXCL1/CXCR2/NF-κB signaling pathway.

Our study has few limitations. Firstly, our study focused only on the CXCL1/CXCR2/NF-κB signaling pathway. Secondly, we identified CXCL1 mRNA as the only target of miR-532-5p in CI/RI. Therefore, future investigations are needed to identify other critical signaling pathways and target mRNAs of miR-532-5p in CI/RI.

In conclusion, miR-532-5p suppressed CI/RI progression by targeting CXCL1. Moreover, our results suggest that miR-532-5p overexpression may be therapeutically beneficial for ischemic stroke patients.

## MATERIALS AND METHODS

### Cell culture

Human neuroblastoma cell (SH-SY5Y) was obtained from American Type Culture Collection (ATCC, Rockville, MD, USA), and and rat adrenal medulla pheochromocytoma cell lines (PC-12) were obtained from Chinese Academy of Sciences (Shanghai, China). All cells were cultured in Dulbecco’s Modified Eagle’s Medium (DMEM, Thermo Fisher Scientific, Waltham, MA, USA) with 10% FBS (Thermo Fischer Scientific) and 1% penicillin and streptomycin (Sigma Aldrich, St. Louis, MO, USA) in the condition of 37°C, 5% CO_2_, and 95% O_2_.

### Cell transfections

SH-SY5Y cells were transfected with negative control (NC) or miR-532-5p agomir (50 nM; both from Ribobio, Guangzhou, China) using Lipofectamine 2000 (Invitrogen, Waltham, MA, USA) for 4 h. Then, the cells were rinsed in warm DMEM medium and further grown for another 24 h. The concentration of agomir was selected. For CXCL1 overexpression, SH-SY5Y cells were transfected with pcDNA3.1 or pcDNA3.1-CXCL1 by using Lipofectamine 2000 (Invitrogen, Waltham, MA, USA) for 24 h. pcDNA3.1 and pcDNA3.1-CXCL1 were purchased from Genepharma (Shanghai, China).

### OGD/R treatment

SH-SY5Y cells were first cultured in deoxygenated glucose-free DMEM medium using a hypoxic vessel maintained at 37°C for 4 h (95% N_2_ and 5% CO_2_). Then, the cells were transferred onto high glucose DMEM medium containing 10% FBS and cultured under normoxic conditions (5% CO_2_) at 37°C for 24 h as previously described [33].

### Quantitative real time polymerase chain reaction (RT-qPCR)

TRIzol reagent (TaKaRa, Tokyo, Japan) was applied to isolate total RNA from cell lines or brain tissues. Then, we reverse transcribed total RNA samples into cDNA using the reverse transcription kit (TaKaRa, Ver.3.0). Real-Time qPCR was then performed in triplicates using the following protocol: 2 minutes at 94°C, followed by 35 cycles of 30 seconds at 94°C and 45 seconds at 55°C. The qPCR primers were obtained from GeneCreate Biological Engineering Co., Ltd (Wuhan, China), and were as follows:

MiR-532-5p forward: 5′-TCACAGTGGCTAAGTTCC GC-3′;

MiR-532-5p reverse: 5′-CTCAACTGGTGTCGTGGA GTC-3′;

CXCL1 forward: 5′-CAAAATGATGAACCCCAGC TC-3′;

CXCL1 reverse: 5′-CATCCTACCATAGCCATTGC AG-3′;

β-actin forward: 5′-TGAAGGGTGGAGCCAAAAG-3′;

β-actin reverse: 5′-AGTCTTCTGGGTGGCAGTGAT-3′;

U6 forward: 5′-CTCGCTTCGGCAGCACAT-3′;

U6 reverse: 5′-AACGCTTCACGAATTTGCGT-3′;

MiR-532-5p and CXCL1 levels relative to U6 and β-actin levels, respectively, were quantified performing the 2^−ΔΔCt^ method.

### CCK-8 assay

The control (NC) or miR-532-5p agomir-transfected SH-SY5Y cells (5 × 10^3^ per well) were seeded overnight and then treated with normoxia or OGD/R for 48 h. Then, we added 10 μl CCK-8 reagent (Beyotime, Shanghai, China) into each well and incubated the cells for another 2 h at 37°C. Finally, we measured the absorbance at 450 nm using a microplate reader (Thermo Fisher Scientific).

### Cellular apoptosis

The control or miR-532-5p-transfected SH-SY5Y cells were treated with normoxia or OGD/R. Then, they were trypsinized, washed with PBS, resuspended and stained with 5 μl Annexin V and propidium (PI) in the dark for 15 min. The stained cells were analyzed in a BD flow cytometer (BD, Franklin Lake, NJ, USA) and the proportion of apoptotic cells (Annexin V^+^ PI^+^ and Annexin-V^+^ PI^−^) were analyzed using a FACS software.

### Estimation of intracellular reactive oxygen species

After treatments, SH-SY5Y cells were incubated in buffer containing ROS-sensitive dye, DCFDA (Beyotime, Shanghai, China) as previously described [34] for 20 min. Then, the cells were centrifuged at 300 x*g*, washed and resuspended in 1X PBS and analyzed by FACS using a BD flow cytometer (BD, Franklin Lake, NJ, USA). The relative levels of ROS were measured based on DCFDA intensity with appropriate controls.

### Enzyme-linked immunosorbent assay (ELISA)

IL-1β, IL-6, TNF-α, and MPO levels in the supernatants of SH-SY5Y cells were measured using ELISA kits from ELK Biotech (Wuhan, China). LDH, MDA, and SOD levels in SH-SY5Y cell supernatants were tested using ELISA kits (Nanjing Jiancheng Bioengineering Institute, Nanjing, China).

### Western blotting

The RIPA buffer was performed to prepare the total protein lysates from SH-SY5Y cells or rat tissues, and the total protein was quantified with the BCA protein assay kit (Beyotime, Shanghai, China). Then, protein samples were separated on 10% SDS-PAGE and transferred onto PVDF membranes (Bio-Rad, Hercules, CA, USA). The membranes were blocked with 5% skimmed milk in TBST for 1 h. Then, the membranes were incubated with primary antibodies, namely, anti-CXCL1 (Abcam, Cambridge, MA, USA; 1:1000), anti-CXCR2 (Abcam; 1:1000), anti-p65 (Abcam; 1:1000), anti-p-p65 (Abcam; 1:1000) and anti-β-actin (Abcam; 1:1000) at 4°C overnight. Subsequently, the membranes were incubated with the secondary anti-rabbit antibody (Abcam; 1:5000) at room temperature for 1 h. The membranes were then developed with ECL, scanned using an Odyssey Imaging System, and analyzed with Odyssey v2.0 software (LICOR Biosciences, Lincoln, NE, USA) to determine protein band intensity. The levels of CXCL1, CXCR2, p65, and phospho-p65 were measured relative to β-actin, which was used as an internal loading control.

### Dual luciferase reporter assay

We cloned wild type (wt) or mutant (mt) 3’UTR of CXCL1 into the pmiRGLO vector (Promega, Fitchburg, WI, USA) and designated them as wt-CXCL1 or mt-CXCL1, respectively. Then, SH-SY5Y cells were co-transfected with miR-532-5p mimics and wt-CXCL1 or mt-CXCL1 using Lipofectamine 2000. After 48 h, relative luciferase activity was measured using the dual-luciferase reporter assay system (Promega).

### 5-aza-2'-deoxycytidine (5-Aza) treatment

SH-SY5Y cells were grown in DMEM containing 5 μmol/L 5-Aza-2’-deoxycytidine (5-Aza; Sigma-Aldrich, CA, USA) for 72 h. The DMEM with 5-Azacytidine was replaced every 24 h. Then, total RNA was extracted from exponentially growing control and 5-Aza-treated cells and subjected to RT-qPCR and methylation-specific PCR (MSP).

### MSP

For MSP detection, we performed PCR with a pair of primers that amplify methylated or unmethylated CpG island sequences of the miR-532-5p promoter. The methylation-specific primers were as follows: 5′-TTATTTGTGGTAGAATTTTGGCG-3′ (forward) and 5′-CATATTAATCCCAATACATACGTCG-3′ (reverse). The primers that amplified unmethylated CpG island sequences of the miR-532-5p promoter were: 5′-GATTATTTGTGGTAGAATTTTGGTG-3′ (forward) and 5′-ACATATTAATCCCAATACATACATCAC-3′ (reverse). The PCR products were resolved by agarose gel electrophoresis and stained with GoldView I (Solarbio, Beijing China).The information of the MSP-amplified region is shown in the Supplementary material.

### *In vivo* MCAO model rats

Forty eight Wistar rats (aged 6 weeks, Chinese Academy of Sciences, Shanghai, China) were purchased from Vital River (Beijing, China) and housed in a dedicated SPF facility. The rats were randomly divided into four groups (*n* = 12 per group): (1) sham-operated (Sham); (2) miR-532-5p agomir; (3) I/R (MCAO), and (4) I/R plus miR-532-5p agomir. The rats in groups 3 and 4 were anesthetized with 45 mg/kg pelltobarbitalum natricum. Then, focal cerebral ischemia was induced by MCAO treatment as previously described [35]. The filament was removed after 1 h and reperfusion was performed. The rats in groups 1 and 2 suffered the same protocol, while it needs no filament insertion. We then injected saline by intra-ventricular injections into rats belonging to groups 1 and 3 , whereas, rats belonging to groups 2 and 4 received intra-ventricular injection containing miR-532-5p agomir as previously described [36]. The rats were sacrificed after 3 days. Then, we harvested the whole brain tissues and determined the cerebral infraction area, water content and other parameters. The animal experiments were performed in line with the NIH guidelines for the care and use of laboratory animals and the protocol approved by the Ethics Committees of Southeast University (No. SU20190507).

### TUNEL staining

The paraffin sections were permeabilized and treated with 50 μl TUNEL reaction mixture (Sigma Aldrich, St. Louis, MO, USA) for 60 min at 37°C with no light. Then, the slides were treated with 50 μl peroxidase (POD, Beyotime) for 30 min, followed by incubation with diaminobenzidine substrate solution (50 μl, DAB, Beyotime) for 10 min at 25°C. The TUNEL-positive apoptotic cells in the brain tissues were analyzed using an optical microscope (Tokyo, Japan).

### Brain water content detection

Briefly, the cerebral hemispheres were separated quickly after harvesting the rat brains and their wet weights were assessed. Then, the cerebral hemispheres were dried at 120°C for 48 h and the dry weight was estimated. The water content of the brain was calculated as follows: [(wet weight-dry weight)/wet weight] × 100%.

### 2, 3, 5-Triphenyltetrazolium chloride (TTC) staining

In brief, brain tissues from the four different rat groups were frozen for 30 min. Then, the brain tissues were cut and treated with 2% TTC solution (Sigma) for 20 min. Subsequently, the sections were fixed and photographed under a light microscope. The cerebral infarction area was expressed as follows: total infarct volume/total brain volume × 100%.

### Statistical analysis

The data are expressed as means ± standard deviation (SD) of at least three independent experiments (12 rats per group in animal study). The data between two groups was analyzed using Student’s *t*-test. The differences between multiple groups (≥ 3) were analyzed by one-way analysis of variance (ANOVA) followed by Tukey’s test. *P* < 0.05 means statistically significant. Graphpad prism version 8.0 was used for the statistical analysis.

## Supplementary Materials

Supplementary Figure 1
