# Supplementary material for: MicroRNA-532-5p protects against cerebral ischemia-reperfusion injury by directly targeting CXCL1
Source: Aging (Albany NY). 2021 Apr 18;13(8):11528–41. doi: 10.18632/aging.202846 (PMC8109118; doi:10.18632/aging.202846)
Supplement: Supplementary Figure 1 [file aging-13-202846-s001.pdf]

SUPPLEMENTARY FIGURE

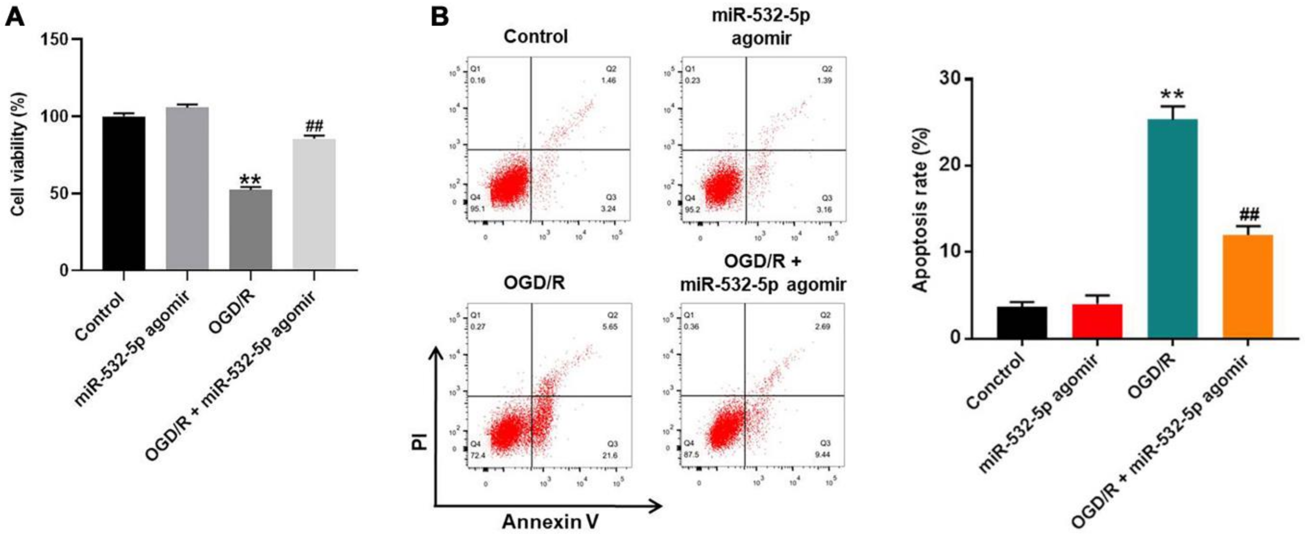

Supplementary Figure 1. OGD/R-induced PC-12 cell growth inhibition was reversed by miR-532-5p agomir. (**A**) PC-12 cells were treated with miR-532-5p agomir, OGD/R or OGD/R plus miR-532-5p. Then, the viability of PC-12 cells was tested by CCK-8 assay. (**B**) The apoptosis of PC-12 cells was tested by flow cytometry. $^{**}P < 0.01$ compared to Control; $^{\#\#}P < 0.01$ compared to OGD/R. All experiments were performed in triplicates.
